# Immune Regulation and Antitumor Effect of TIM-1

**DOI:** 10.1155/2016/8605134

**Published:** 2016-06-20

**Authors:** Peng Du, Ruihua Xiong, Xiaodong Li, Jingting Jiang

**Affiliations:** ^1^Department of Tumor Biological Treatment, The Third Affiliated Hospital, Soochow University, Changzhou, Jiangsu 213003, China; ^2^Jiangsu Engineering Research Center for Tumor Immunotherapy, Changzhou, Jiangsu 213003, China; ^3^The Second People's Hospital of Gansu Province, Lanzhou, Gansu 730000, China; ^4^Department of Oncology, The 181st Hospital of PLA, Guilin, Guangxi 541002, China

## Abstract

T cells play an important role in antitumor immunity, and the T cell immunoglobulin domain and the mucin domain protein-1 (TIM-1) on its surface, as a costimulatory molecule, has a strong regulatory effect on T cells. TIM-1 can regulate and enhance type 1 immune response of tumor association. Therefore, TIM-1 costimulatory pathways may be a promising therapeutic target in future tumor immunotherapy. This review describes the immune regulation and antitumor effect of TIM-1.

## 1. Introduction

Immune suppression is an important factor for immune evasion of tumor. Generally, the immune systems of tumor patients often have excessive inhibitory functions, which are induced by regulatory T cells (Tregs), myeloid-derived suppressor cells (MDSCs), or the secretion of immunosuppressive cytokines, such as tumor growth factor-*β* (TGF-*β*) and interleukin-10 (IL-10). These conditions constitute an extremely favorable microenvironment for tumor progression [1–4]. Therefore, it is important to find novel targets for reversing immunosuppression microenvironment.

The identification of new classes of costimulatory molecules provides new exciting opportunities for inducing and enhancing effective endogenous immune response to cancer. TIM-1, a key member and costimulatory molecule in the T cell immunoglobulin mucin (TIM) family, is expressed on the surface of T cells. It can promote the activation and proliferation of T cells and the secretion of cytokines, which play critical roles in tumor immunity [5–9]. Our preliminary studies have shown that TIM-1 may be a novel candidate tumor therapeutic costimulatory molecule, because it may directly enhance the functions of CD8^+^ T cells and/or NK cells, as well as altering the tumor microenvironment for more effective antitumor immune response (data not shown). This review tries to describe how TIM-1 regulates immune function and takes part in antitumor immune responses and illustrates the mechanism of immune regulation.

## 2. Structure and Basic Function of TIM-1

In human, there are three members (TIM-1, TIM-3, and TIM-4) located in the human chromosome 5q33.2 region. In mouse, the TIM family consists of eight members (TIMs 1–8) located in the 11B1.1 region of chromosome. The human and mouse TIM family genes are highly homologous [8, 10]. Like other TIM members, TIM-1 is similar in structure to the type 1 membrane protein, which consists of an N-terminal Cys-rich immunoglobulin variable- (IgV-) like domain, a mucin-like domain, a transmembrane domain, and an intracellular tail [11, 12]. The intracellular tail of TIM-1 contains tyrosine phosphorylation motifs that are involved in transmembrane signal [8, 13–15].

The expression of human TIM-1 was first detected in damaged kidney and named human kidney injury molecule-1 (KIM-1) [16–19]. Previous studies have indicated that* in vivo* TIM-1 gene mutations in human and mouse are associated with some allergic diseases [8, 20]. Abnormal expression of TIM-1 is related to some autoimmune diseases [21–27]. In recent years, study found that TIM-1 is mainly expressed on the surfaces of CD4^+^ T cells, CD8^+^ T cells, NK cells, macrophages, DCs, B cells, and mast cells [28]. Moreover, it is also found that TIM-1 is expressed in lymphoid tissues [8, 29] and confirmed that TIM-1 can promote the production of cytokines and enhance the antigen induced immune response of T cells [30–35]. Therefore, TIM-1 may be a potential costimulatory molecule to enhance antitumor immune response [8, 23, 35–38].

## 3. Immune Regulation of TIM-1

TIM-1 is a highly efficient costimulatory molecule, which can enhance the formation of CD3-TCR with agonistic anti-TIM-1 antibody involved in the activation of T cells [7, 8, 37, 39]. The main ligands of TIM-1 are TIM-4 and phosphatidylserine (PS) [36, 40, 41]. TIM-4 is expressed on the surface of antigen presenting cells (APCs) such as macrophages and dendritic cells, working as an endogenous ligand of TIM-1 [5, 42, 43]. TIM-4 can promote T cell activation, proliferation, and cytokine production by binding to TIM-1, which mediates the positive regulation of T cells and triggers the immune response with costimulatory effect [30, 40]. PS is another important ligand of TIM-1 and can activate NKT cells by binding to TIM-1 on the surface of NKT cells [12, 44, 45]. In addition, P-selectin and S-selectin are also potential ligands for TIM-1 and may play roles in inflammation and autoimmune diseases. This signal pathway is closely related to the migration of Th1 and Th17 cells in blood vessels [38, 46].

The biological function of TIM-1 mainly depends on lymphocytes. TIM-1 in CD4^+^ T cells can upregulate the activation signal of T cells by interacting with T cell receptor (TCR), which promotes the synergistic effect of TIM-1 [8, 47]. In immune regulation, the positive and negative regulation of TIM-1 are essential for the maintenance of immune homeostasis. The immune regulation of TIM-1 mainly depends on its ligands [8]. It has been reported that agonistic TIM-1 mAbs (clone 3B3 and clone 1H8.2) augment T cell-mediated immune responses, whereas an antagonistic antibody inhibits immune responses through regulatory B cells [48]. Agonistic TIM-1 monoclonal antibody can promote the proliferation of CD8^+^ T cells* in vitro* and enhance their biological function [49]. The different effects of agonistic and antagonistic TIM-1 mAbs* in vivo* may be due to the fact that different TIM-1 mAbs deliver qualitatively and quantitatively different signals to T cells and B cells. The TIM-1 signaling on B cells is important in maintaining normal homeostasis of the immune system and preventing systemic autoimmunity [50, 51]. In CD4^+^ T cells, the TIM-1 molecules bound with agonistic TIM-1 mAbs [39] or other agonistic ligands can produce a strong costimulation signal to activate T cells, promote the differentiation and proliferation of T cells* in vivo*, activate the production of cytokines, and enhance the antigen induced immune response of T cells [30–34]. Previous studies have found that the inhibition of TIM-1 signal of CD4^+^ T cell can reduce the level of white blood cells and the production of inflammatory mediators, which can reduce the tissue damage caused by excessive inflammatory reactions [30, 35, 52, 53].

The negative regulation of immune function of TIM-1 in B cells plays a key role in preventing immune rejection [51, 54]. The inhibition of TIM-1-Fc signaling inhibits the differentiation and function of CD4^+^ T cells and further reduces chronic rejection reactions [55]. Zhang et al. have found that the suppression of the TIM-1 signal in CD4^+^ T cells can inhibit the activity of macrophages and reduce the injury of transplanted liver in a mouse model [56]. TIM-1 is also a key molecule in the regulation of immune rejection of allogeneic transplantation [49], and functional deficiency of TIM-1 is also one of the mechanisms of autoimmune diseases [50]. The expressions of TIM-3 and TIM-1 on the surface of mouse mast cells promote the secretion of IL-13, IL-6, and IL-4, indicating that mast cells also regulate immune function through TIM members [57]. Study also found that the inhibition of TIM-1 signal can reduce infiltration of T cells into allergic skin tissues and tissues of autoimmune diseases [38], and deficiency of TIM-1 reduces the incidence of allergic asthma in a mouse model [58]. Therefore, TIM-1 may also be related to the molecular mechanism of allergic diseases.

## 4. TIM-1 for Cancer Immunity

Type 1 immune response, mediated by Th1 cells, cytotoxic T lymphocytes (CTLs), NK cells, NKT cells, and gamma delta T cells, is considered as a critical component of cell-mediated immunity against tumor. CD8^+^ T cells are important T cell subsets in specific immune response. They are the final effector cells to kill tumor and inhibit tumor progression* in vivo*, which are widely used in tumor adoptive immunotherapy [59, 60]. In human, the presence of Th1 cells and CTLs in tumor can be a favorable prognostic indicator [61]. However, many tumor infiltrating Th1 and CD8^+^ T cells are in a status of nonresponsiveness due to local and systemic mechanisms of immune suppression in cancer patients as well as in tumor-bearing mice and even play a protective role for tumor [62, 63]. The lack of costimulation of type 1 lymphocytes is the major mechanism underlying tumor-induced immune tolerance [64, 65]. Thus, agonistic antibodies against costimulatory receptors such as 4-1BB and CD40 have shown promising antitumor effects in various preclinical tumor models, which are evaluated in clinical trials. The costimulation signal plays an important role in CD8^+^ T cells [64]. In the model of acute renal injury induced by cisplatin, blocking of TIM-1 signal can significantly reduce the number of CD8^+^ T cells and inhibit the secretion of IFN-*γ*, indicating that TIM-1 costimulation signal can enhance the effect of CD8^+^ T cells [66].

In the TIM family, to date, it has been confirmed that TIM-3 is related to tumor [67, 68] and found that the expression of TIM-3 has an important influence on tumor microenvironment [69, 70]. However, we still have a lot of unknowns regarding the effects of tumor immunity of TIM-1. There are only a few articles that can be retrieved, which are about antitumor effect of TIM-1 [5, 6], but it has been determined that TIM-1 can promote the proliferation and differentiation of T cells by binding to different agonistic ligands [15, 30, 40, 71]. A study has demonstrated that TIM-1 tyrosine phosphorylation can recruit the PI3K adaptors p85, which stimulates the activation and function of T cells [15]. In tumor microenvironment, the effector cells, such as CD8^+^ T cells, directly participate in immune response and can enhance antigen recognition, proliferation, and differentiation of other effector cells.

Ligation of the transmembrane protein TIM-1 can costimulate T cell activation by the PI3K signaling pathway. Agonistic antibodies to TIM-1 are also capable of inducing T cell activation without additional stimuli; PI3K is an important factor in mediating TIM-1 signaling [15]. It has been known that the PI3K/Akt/mTOR signaling pathway plays a crucial role in the regulation of cell growth, proliferation, and metabolism. The immune cells and tumor cells compete for energy. The activation of some signaling molecules closely related to energy metabolism regulates T cell activation, differentiation, and function and further enhances the antigen recognition, proliferation, and the differentiation of T cells. So far, PI3K/Akt/mTOR signaling pathway is a target of tumor therapy [72–77].

**Figure 1:**
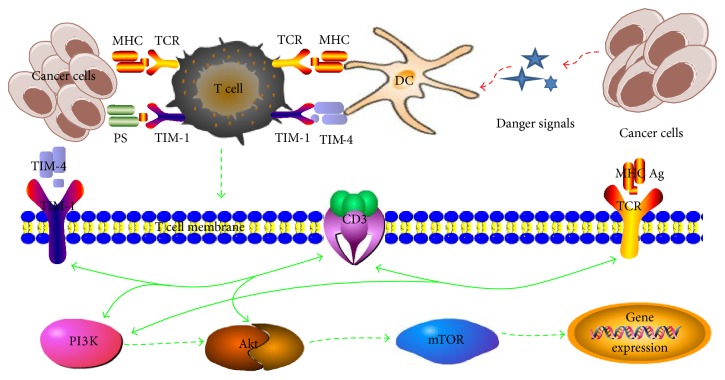
Tumor cells release signals, which are received by dendritic cells (DCs). Tumor antigens are processed to MHC antigens and then presented to the T cell receptor (TCR) for activation. TIM-4 (or phosphatidylserine) on DCs binds to TIM-1 on T cells to form the CD3-TCR complex, which participates in TCR-mediated T cell activation and initiates the intracellular PI3K signal pathway. PI3K signal pathway consists of the interaction between TIM-1 and ligands, tyrosine phosphorylation of the intracellular region of TIM-1, the recruitment of PI3K, the activation of Akt by PI3K, and the activation of mTOR by Akt. Activated mTOR can regulate the biological functions of T cells.

The transcription factor T-bet/Eomes is involved in the regulation of CD8^+^ T cell function and induces the differentiation of CD8^+^ T cells to effector and central memory T cells [78, 79]. The expression level of TIM-1 and T-bet/Eomes has important effects on regulating the biological function of T cells, and the expression of T-bet is closely related to the prognosis of tumor patients [24, 80]. We have analyzed 152 cases of gastric cancer patients and found that the expression of T-bet is closely related to the survival of tumor patients. The number of T-bet positive T cells in tumor tissues has a significant effect on the prognosis of the patients [81]. T-bet/Eomes, which stimulates the activation and differentiation of CD8^+^ T cells, is significantly upregulated in the tumor of the third day after radiofrequency ablation (RFA), and the expression level of TIM-1 in infiltrating CD8^+^ T cells is significantly upregulated. In T-bet/Eomes double knockout tumor model mice, it has been found that the expression of TIM-1 is very low in infiltrating CD8^+^ T cells stimulated by tumor antigen, and in wild type mice it is significantly upregulated (data not shown). At present, TIM-1 is considered to improve the secretion of some cytokines such as IL-4 and IFN-*γ* [82]. Type 1 immune response of TIM-1-mediated T cell activation is associated with tumor immunity through transcription factor T-bet/Eomes [71, 83] and the PI3K signal pathway [15] (Figure 1).

## 5. Prospect

We speculate that TIM-1, a new costimulatory candidate molecule for tumor treatment, not only directly enhances the antitumor effect of CD8^+^ T cells and NK cells but also changes the tumor microenvironment to induce more effective antitumor immune response. As a target molecule, it may have a good application prospect in clinical cancer research. In addition, agonistic anti-TIM-1 monoclonal antibody or other ligands can enhance the function of T cells [39, 82], increase CD8^+^ T cells and NK cells, reduce MDSC in tumor tissues, and inhibit tumor growth (data not shown). It is important to define the mode of action and determine whether CD8^+^ T cells and NK cells mediate the antitumor effect of agonistic TIM-1 mAbs* in vivo*. These may provide a theoretical basis to construct a new tumor therapy model of TIM-1 signal interference.
